# Targeting the NG2/CSPG4 Proteoglycan Retards Tumour Growth and Angiogenesis in Preclinical Models of GBM and Melanoma

**DOI:** 10.1371/journal.pone.0023062

**Published:** 2011-07-29

**Authors:** Jian Wang, Agnete Svendsen, Justyna Kmiecik, Heike Immervoll, Kai Ove Skaftnesmo, Jesús Planagumà, Rolf Kåre Reed, Rolf Bjerkvig, Hrvoje Miletic, Per Øyvind Enger, Cecilie Brekke Rygh, Martha Chekenya

**Affiliations:** 1 Translational Cancer Research Group, Department of Biomedicine, University of Bergen, Bergen, Norway; 2 Haukeland University Hospital, Department of Pathology, Bergen, Norway; 3 The Gades Institute, University of Bergen, Bergen, Norway; 4 Heart and Circulatory Research Group, Haukeland University Hospital, Bergen, Norway; 5 Centre de Recherche Public de la Santé, Luxembourg, Luxembourg; 6 Department of Neurosurgery, Haukeland University Hospital, Bergen, Norway; Sun Yat-sen University Medical School, China

## Abstract

Aberrant expression of the progenitor marker Neuron-glia 2 (NG2/CSPG4) or melanoma proteoglycan on cancer cells and angiogenic vasculature is associated with an aggressive disease course in several malignancies including glioblastoma multiforme (GBM) and melanoma. Thus, we investigated the mechanism of NG2 mediated malignant progression and its potential as a therapeutic target in clinically relevant GBM and melanoma animal models. Xenografting NG2 overexpressing GBM cell lines resulted in increased growth rate, angiogenesis and vascular permeability compared to control, NG2 negative tumours. The effect of abrogating NG2 function was investigated after intracerebral delivery of lentivirally encoded shRNAs targeting NG2 in patient GBM xenografts as well as in established subcutaneous A375 melanoma tumours. NG2 knockdown reduced melanoma proliferation and increased apoptosis and necrosis. Targeting NG2 in two heterogeneous GBM xenografts significantly reduced tumour growth and oedema levels, angiogenesis and normalised vascular function. Vascular normalisation resulted in increased tumour invasion and decreased apoptosis and necrosis. We conclude that NG2 promotes tumour progression by multiple mechanisms and represents an amenable target for cancer molecular therapy.

## Introduction

GBM is the most common and aggressive brain tumour in adults, where the median survival after diagnosis is only 14.6 months [1], [2], [3], despite advances in multimodal therapeutic options. This is partly due to diffuse invasion that invariably results in tumour recurrence, molecular and cellular heterogeneity that renders them therapy resistant. GBMs are characterised by florid angiogenesis, formed by leaky and variably perfused chaotic vasculature that results in tissue necrosis and high tumour interstitial pressure that further prevents the entry of cytotoxic agents. Thus, there is a major need for the development of local therapies that target the cell types that mediate this aggressive disease course.

We have previously shown that a subpopulation of GBM cells express Neuron-glia 2 (NG2/CSPG4), also known as melanoma proteoglycan (MPG), a cell surface chondroitin sulphate proteoglycan that is also expressed by numerous tissue specific progenitor cells during development. The *CSPG4* gene encoding the NG2 proteoglycan, is turned off upon terminal differentiation, but is aberrantly re-expressed by several tumour types [4], [5], [6], [7], [8], [9]. Shoshan and co-workers demonstrated NG2 expression in one out of five GBMs by immunoblotting [8], whilst we showed moderate to high expression in 6 out of 14 GBM biopsies from the tumour mass and confrontation edge by immunohistochemistry [5]. The discrepancies in these two studies are likely explained by the method of analysis and the small sample sizes. In agreement with our previous results, we recently demonstrated in 74 GBM biopsies that NG2 was highly expressed on tumour cells and angiogenic vessels in 50% of GBM patients, and was associated with significantly shorter survival outcomes. This effect of NG2 on poor survival was independent of age at diagnosis, treatment received and hypermethylation of the O^6^-methylguanine methyltransferase (*MGMT*) DNA repair gene promoter (Svendsen et al., unpublished data). The association of high NG2 expression with an aggressive disease course may be mediated by its role in increasing cell proliferation, angiogenesis and treatment resistance[10], [11], [12].

Others have demonstrated high NG2 expression in 95% of uveal melanomas [13], that it increased the metastatic potential of melanomas, and soft tissue sarcomas [14], [15], and mediated a poor prognosis in childhood acute myeloid leukaemia (AML) patients[9]. Since NG2 is expressed on the surface of both tumour cells and pericytes, it is an attractive candidate for simultaneously targeting the malignant and stromal cellular compartments within the tumour.

Several treatment approaches, such as anti-angiogenesis treatment and viral gene therapies [16], [17] have shown promising results in preclinical models. However, only a fraction of the drug candidates that show good results in animal studies is verified to have a treatment effect in patients [16], [18], [19]. This lack of correlation between effects observed in pre-clinical models and in patients is largely due to limited cellular heterogeneity in syngeneic animal tumours, smaller tumour burden due to their smaller brains, altered growth characteristics [20], [21], lack of invasive tumour cells in most tumour models, and lack of appropriate antigen profiles compared to human brain tumours. A novel animal model of brain tumours has been developed in our laboratory where patient GBM biopsies minimally propagated *in vitro* as spheroids [20], [21], [22] are implanted intracranially into athymic rats[23]. As opposed to the well circumscribed, non-invasive tumours established from GBM cell-lines, xenografts established from tumour biopsy-spheroids show striking histological and genetic features of the patient GBMs *in situ*, such as diffuse invasion of the brain parenchyma, and cellular heterogeneity [22]. The aims of the present study were to investigate the mechanisms of NG2 mediated malignant progression in multiple tumour models and validate it as a cancer therapeutic target. We asked whether abrogation of NG2/CSPG4 function with shRNAs inhibited tumour growth in human GBM and melanoma xenografts.

## Results

### NG2 overexpressing GBM tumours share characteristics of patient GBM

**Figure 1:**
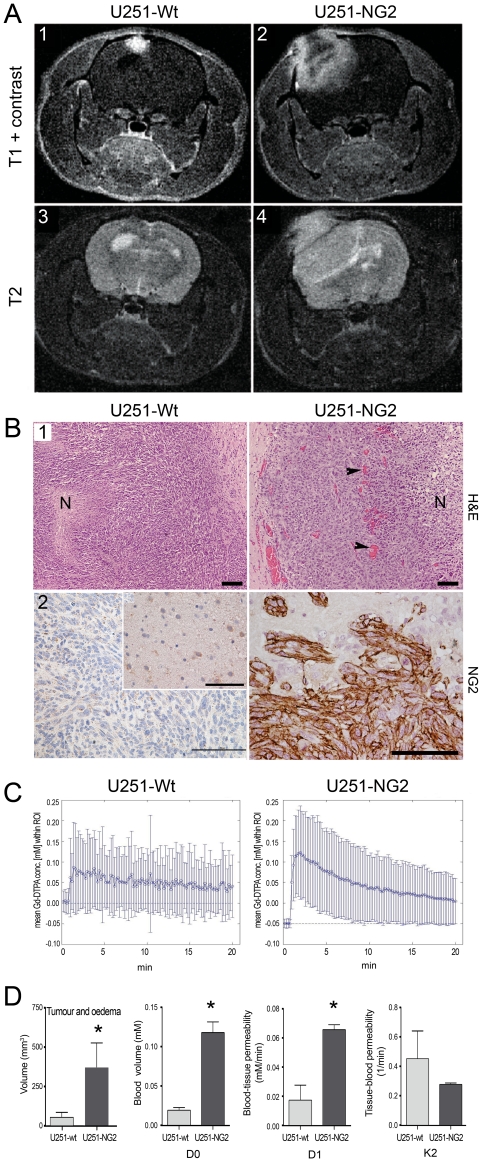
Growth characteristics of NG2 negative and positive tumours. The upper panels show post-contrast T1-weighted MRI images of U251-wt (A_1_), and U251-NG2 tumours (A_2_) and the corresponding T2 weighted images of U251-wt (A_3_) and U251-NG2 tumours (A_4_). (B) H&E Comparisons of NG2 U251-Wt (B1, left panel) and U251-NG2 GBMs showing numerous leaky blood vessels, pleomorphic tumour cells and necrosis (B_1_, right panel). In the NG2 negative U251-Wt tumours (B_2_, left panel), expression was observed only on oligodendrocyte progenitors in normal brain (B_2_, insert), Invasive NG2 positive U251-NG2 tumour cells (B_2_, right panel). (C) Vascular parameters determined from pharmacokinetic models of tracer concentration/time curves where the initial step and slope of the curve is representative of D0 (fractional blood volume)_,_ and D1 (blood-tissue permeability), and the decay phase is representative of K2 (washout of tracer). Significant increased tumour and vasogenic oedema volumes of U251-NG2 tumours compared to U251-wt tumours (D, *p<0.05). Significant elevation of D1 (D, Mann Whitney U Test; *p = 0.0317) and D0 in the U251-NG2 tumours (D, Mann Whitney U Test; *p = 0.0159) compared to U251-wt tumours. K2 levels in the NG2 positive and negative tumours (D, Mann Whitney Test; p = 0.73). Scale bars in B_1_ = 100 µm, magnification 100×; scale bars in B_2_ = 100 µm, magnification 200×. N denotes necrosis; arrowheads: large vessels “vascular lakes”.

To investigate the significance of NG2 on tumour growth, U251 GBM cells overexpressing its gene *CSPG4*, were xenografted into nude rat brains and tumour progression compared to NG2 negative xenografts. NG2 expression enhanced the growth of the tumours compared to the NG2 negative controls (Fig. 1) that were smaller and showed a homogeneously high signal intensity on T1 weighted MRI after contrast agent injection (Fig. 1A_1_). The NG2 positive tumours were larger, exhibited MRI detectable central necrosis and displayed similar characteristics to human GBMs *in situ* (Fig. 1A_2_). Only obstructive cerebrospinal fluid (CSF) was apparent in the NG2 negative tumours (Fig. 1A_3_), while peri-tumoural oedema was most evident in the rapidly growing NG2 positive tumours, as shown by T2 weighted MRI (Fig. 1A_4_). Indeed, quantification of the solid tumour tissue and oedema revealed a significantly slower growth rate of the NG2 negative tumours, with mean volumes approximately 25% of those of NG2 positive tumours (Fig. 1D). NG2 expression resulted in more necrosis, numerous and dilated vessels (Fig. 1B_1_, right panel, arrowheads), in contrast to the NG2 negative tumours (Fig. 1B_1_, left panel). Examination of the invasive edges in these tumours revealed microsatellite invasion of the brain parenchyma by positive NG2 tumour cells. U251-Wt tumours were uniformly NG2 negative (Fig. 1B_2_, left panel), except for expression on oligodendrocyte progenitors in normal brain (Fig. 1B_2_, insert). To clarify whether the alterations in vascular morphology were also accompanied by functional changes, we estimated microvascular parameters such as permeability, fractional blood volume and elimination rate of contrast agent, as well as the extent of vasogenic oedema. There was a significant increase in tumour and oedema volumes in the U251-NG2 compared to the U251-wt tumours (Fig. 1D left; p<0.05) indicating rapid growth and increased oedema. The vascular parameters were determined from pharmacokinetic models of tracer concentration/time curves where the initial up-slope of the curve is representative of D0_,_ the peak of the curve correlates to (D1), and the decay phase of the curve is representative of the (K2) (wash out of the tracer; Fig. 1C). Indeed, blood-tissue permeability (D1) was significantly elevated in the NG2 positive tumours, (Fig. 1D; Mann Whitney Test; p = 0.0317) which is also consistent with the increased oedema. Likewise, the fractional blood volume, (D0), which is a measure of angiogenesis, was also significantly elevated in the NG2 positive tumours (Fig. 1D; Mann Whitney Test; p = 0.0159). However, there was no significant difference in the washout of tracer (K2) from tumour tissue of NG2 positive and negative tumours (Fig. 1D; Mann Whitney Test; p = 0.73). These results are corroborated by similar findings from our previous study [24], in which we compared high and low molecular weight tracers and implemented two different pharmacokinetic models [25]–[26] to quantify tumour vessel permeability for larger molecules.

### The biological effects of NG2 knockdown in patient GBM xenografts

#### Reduction of tumour growth

**Figure 2:**
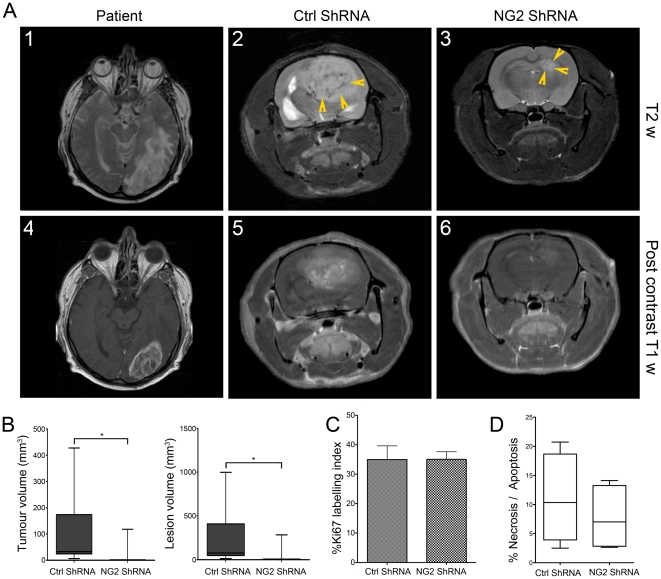
NG2 knockdown in patient biopsy GBM xenografts is associated with reduced tumour volume. MRI of the patient 3: T2 weighted image (A_1_), and T1 weighted image post contrast administration (A_4_). Animals xenografted with P3 biopsy tissue from the same tumour, T2 weighted (A_2_), and post-contrast T1 weighted images (A_5_). MRI of the NG2 shRNA treated animals T2 weighted images (A_3_), and T1 weighted images (A_6_). Tumour (B left boxes, *p = 0.015, t-test) and lesion volumes (B, right boxes, *p = 0.015, t-test) were significantly reduced in the NG2 shRNA tumours compared to the shRNA control tumours. Ki67 labelling in the NG2 shRNA tumours compared to the control shRNA tumours (C, p>0.05). Apoptosis/Necrosis measured by TUNEL staining was reduced in the NG2 knockdown tumours (D, p>0.05). Arrowheads in A_2_ and A_3_ indicate regions of high signal intensity.

To demonstrate that these biological effects were due to NG2 function, we sought to disrupt its expression in GBM biopsy xenografts derived from two different patients using intratumoural delivery of lentivirally encoded shRNAs. We have previously verified that these lentivirally encoded shRNAs successfully abrogate NG2 expression and function [12]. MR imaging of patient 3 (P3) donor revealed a GBM with characteristics typical of the diagnosis, *i.e*. large areas affected by vasogenic oedema illustrated on T2 weighted images (Fig. 2A_1_) heterogeneous contrast enhancement and necrosis on T1 weighted images after administration of gadodiamide (Fig. 2A_4_). The heterogeneity of enhancement reflects the presence of both highly vascularised regions with leaky vessels and necrosis within the same tumour (Fig. 2A_4_). Animals treated with control shRNAs exhibited similar MR features as the patient (Fig. 2A_2_ and 2A_5_) as well as shift of midline structures and obstructive CSF due to the expansive tumour mass (Fig. 2A_2_). The NG2 shRNA treated animals had smaller lesions with minimal MR signal intensity alterations in the tumour compared to NG2 expressing tumours (Fig. 2A_3_ and 2A_6_). The mean tumour volume in the control shRNA treatment group was significantly larger than in the NG2 shRNA treated group, where additionally, not all animals developed MR detectable tumours (*i.e.* 3 out of 8, Fig. 2B left boxes, p = 0.015, two-tailed t-test). High NG2 levels led to increased lesion volumes that included the solid tumour and the peritumoural oedema (Fig. 2B right boxes p = 0.015, Students t-test). However, at e*nd*-stage, there was no significant difference in tumour cell proliferation (Fig. 2C, p>0.05), or apoptosis (Fig. 2D, p>0.05) of the animals treated with control and NG2 shRNAs.

#### Reduction of angiogenesis and increased tumour invasion

**Figure 3:**
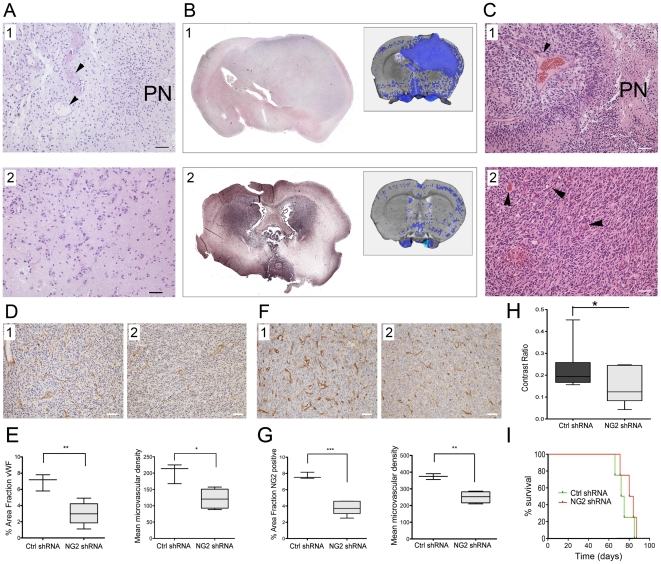
NG2 knockdown in GBM biopsy xenografts reduced angiogenesis and increased tumour invasion. Heterogeneous GBM *in situ* with pleomorphic nuclei, microvascular proliferations (arrowheads) and pseudopalisading necrosis (PN) (A_1_). Patient GBM cells diffusely invading the brain parenchyma (A_2_, scalebar = 100 µm). Expansive, control shRNA treated tumour (B, 1), Inserts: areas with increased T1 weighted signal post contrast overlayed on T2 weighted MR images. NG2 shRNA treated tumours dissemination throughout the brain (B_2_). Pseudopalisading necrosis (PN), and large vessel sprouting (arrowheads) in the shRNA control treated tumours (C_1_) compared to NG2 shRNA treated xenografts (C_2,_ arrowheads), scale bar = 100 µm). Immunostaining for vWF in control shRNA (D_1_) and NG2 shRNA treated tumours (D_2_), scalebar = 100 µm). Quantification of vWF positive area fraction (**p = 0.006, t-test) and microvascular density (*p = 0.010, t-test) in control shRNA compared to NG2 shRNA treated tumours (E, left and right respectively), scale bar = 100 µm). NG2 positive vessels (brown) in control shRNA (F_1_) compared to NG2 shRNA (F_2_) treated tumours (scalebar = 100 µm). Quantification of NG2 positive area fraction (G, left panel, ***p = 0.0003, t-test) and microvascular density (G, right panel, **p = 0.0025 t-test) in control shRNA and NG2 shRNA treated tumours. Contrast ratio between tumour tissue and normal brain in T1 weighted post contrast images in the control and NG2 shRNA treated tumour xenografts (H, *p = 0.032 t-test). Kaplan–Meier survival curves (I, p = 0.3; Log-Rank test).

Characteristically, the P3 GBM was highly heterogeneous with pleomorphic nuclei, microvascular proliferations and regions with pseudopalisading necrosis (Fig. 3A_1_). The invasive edge of the patient biopsy revealed diffuse invasion of tumour cells into the brain parenchyma (Fig. 3A_2_). The histological features of control shRNA and NG2 shRNA treated P3 xenograft tumours were in accordance with the MR images (inserts in Fig. 3B_1_, Fig S1_1_ and videos S1 and S2), showing tumour mass effect that induced shift of the midline structures (determined by ventricular compression and sulcal effacement) (Fig. 3B_1_). The NG2 expressing tumours were highly cellular, contained pseudopalisading necrosis with surrounding pyknotic cells (Fig. 3C_1_) and numerous small and large vessels (Fig. 3D_1_). In contrast, the NG2 shRNA treated tumours frequently exhibited bilateral and microsatellite lesions that did not enhance contrast on T1 weighted images (Fig. 3B_2_, Fig S1_2_ and videos S1 and S2). Despite their dense cellularity, fewer and smaller vessels were detected in these tumours (Fig. 3C_2_ and 3D_2_), where quantification of the vascular area fraction and vascular density proved that the NG2 shRNA treated tumours were significantly less vascularised (microvascular area fraction, p = 0.006 t-test, and microvascular density, p = 0.010 t-test, respectively, Fig. 3E, left and right panels, respectively), compared to the NG2 positive tumours. The area fraction that was NG2 positive was significantly reduced in the NG2 shRNA treated tumours compared control shRNA treated tumours (Fig. 3F_1-2_ and 3G left panel, p = 0.0003, t-test). The number of NG2 positive tumour microvessels was also significantly reduced by the NG2 shRNAs compared to control shRNA treated tumours (Fig. 3F_1-2_ and 3G right panel, p = 0.0025 t-test). The reduction in NG2 positive microvasculature was similar to the reduction in vWF positive vessels in the NG2 shRNA treated tumours. The differences in tumour vasculature were also reflected in the change in contrast enhancement ratio (i.e. the signal intensity ratio between the tumour and healthy brain tissue). Control shRNA treated tumours had a significantly higher contrast ratio between the tumour and brain tissue on T1-wighted MRI images after contrast agent injection (Fig. 3H, p = 0.032), indicating greater vessel permeability compared to the vasculature from NG2 shRNA treated tumours. Although the NG2 shRNA treated animals had a median survival of 82 compared to 73 days of the control shRNA treated animals, this difference was not significant (Fig. 3I, p = 0.3; Log-Rank test).

#### Reduction of haemorrhagic vessels and vasogenic oedema

**Figure 4:**
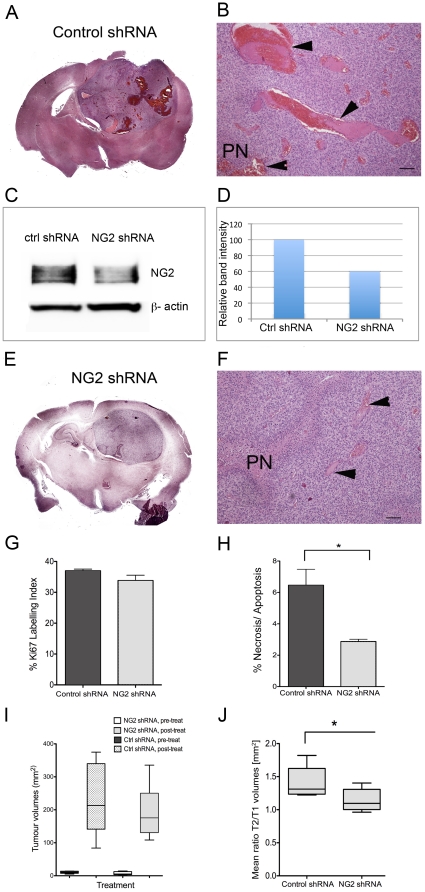
NG2 knockdown in GBM xenografts reduced large, haemorrhagic vessels and vasogenic oedema. H&E stained composite image showing expansive, haemorrhagic and highly angiogenic P13 tumour treated with control shRNA (A). Large leaky vessels with phenotype of “vascular lakes” in the control shRNA treated tumours (B, arrowheads, scale bars = 100 µm). Immunoblot showing NG2 expression after treatment with control shRNA (C) and NG2 shRNAs (D) in P13 tumour cells indicating knockdown. H&E stained composite image showing less vascular and non-haemorrhagic NG2 shRNA treated tumour (E). H&E showing pseudopalisading necrosis (PN), fewer and morphologically smaller vessels after treatment with NG2 shRNAs (F, arrowheads), scale bars = 100 µm). Ki67 labelling in the control and NG2 shRNA treated tumours (G, p>0.05 t-test). % Necrosis/Apoptosis measured by TUNEL staining was reduced in the NG2 knockdown tumours (H, *p = 0.02, t-test). Tumour volumes before and after treatment with control and NG2 shRNAs (I, p>0.05, t-test). The oedema levels measured by the ratio of the total lesion volume (T2 w signal change)/solid tumour volume (T1w post contrast signal change) (J) in the control and NG2 shRNA treated tumours (*p = 0.043).

GBMs are characterized by high intra- and inter-tumoural heterogeneity, hence the moniker “multiforme”. Therefore we sought to validate our findings from P3 by investigating GBM xenografts derived from another patient, (P13) which predominantly expressed NG2 on the tumour vasculature. The tumours treated with control shRNAs were highly angiogenic, indicated by the presence of large, leaky and haemorrhagic vessels, with morphological features of “vascular lakes”, (Fig. 4A and B, arrowheads). In contrast to control shRNAs, treatment with NG2 shRNAs reduced NG2 expression (Fig. 4C and D), and resulted in morphologically smaller and less haemorrhagic vessels (Fig. 4E and DF, arrowheads). Although treatment with NG2 shRNAs did not change the tumour cell proliferation (Fig. 4G), it significantly reduced the amount of cell death (p = 0.02, t-test, Fig. 4H). Treatment with NG2 shRNA had no effect on solid tumour volumes as measured by T1 weighted post contrast MRI, Fig. 4I, or overall survival (p = 0.5, Log-Rank test). However, it significantly reduced levels of vasogenic oedema compared to control shRNA treated tumours as indicated by the ratio of lesion volume (T2-weighted)/solid tumour volume (T1-weighted), p = 0.043, t-test, Fig. 4J.

#### Increased vascular reabsorption, decreased plasma volume and vascular normalization

**Figure 5:**
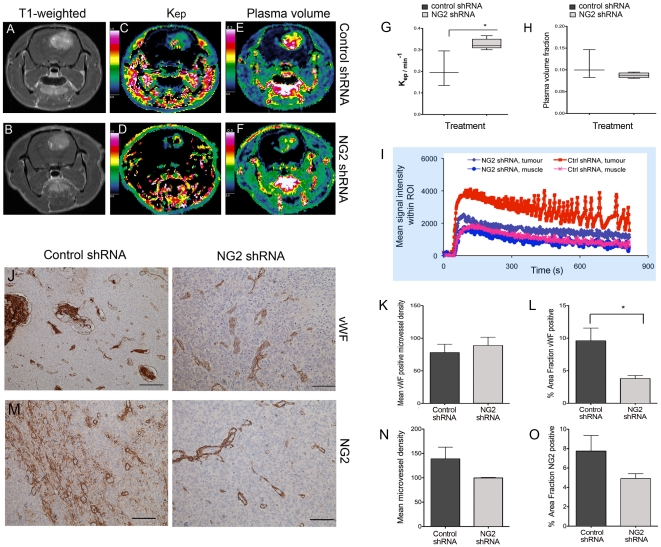
NG2 knockdown in GBM xenografts increased vascular reabsorption, decreased plasma volume and normalized tumour vasculature. Post-contrast T1 weighted images of representative animals xenografted with P13 biopsy tissue and treated with control shRNAs (A), or NG2 shRNAs (B). Kep maps representing efflux of gadodiamide contrast from the extracellular space to the plasma in representative animals treated with control shRNAs (C) and with NG2 shRNAs (D). Fractional plasma volume maps in the same animals treated with control shRNAs (E) and with NG2 shRNAs (F). Quantification of Kep (G, p = 0.018, t-test) and fractional plasma volume (H, p>0.05) in animals treated with control shRNAs compared to NG2 shRNAs. Vascular parameters determined from pharmacokinetic models of gadodiamide induced signal intensity change from baseline/time curves from representative animals in C-F, where the initial step of the curve represents (vascular filling), the slope represents (flow and permeability), the maximum enhancement represents (leakage space) and decay phase represents the washout of tracer (I). Immunostaining for vWF in control shRNA (J, left panel) and NG2 shRNA treated tumours (J, right panel), scalebar = 100 µm). Quantification of vWF positive microvascular density (K, p>0.05) and area fraction (L, p = 0.04, t-test) in control shRNA compared to NG2 shRNA treated tumours, scale bar = 100 µm). NG2 positive tumour cells and vessels in control shRNA treated (M, left panel) compared to NG2 shRNA treated tumours (M, right panel, scalebar = 100 µm). Quantification of NG2 positive microvascular density (N, p>0.05) and area fraction (O, p>0.05) in control shRNA and NG2 shRNA treated tumours, *p<0.05.

The reduction in large haemorrhagic vessels and vasognic oedema after treatment with NG2 shRNAs led us to hypothesise that targeting NG2 normalises vascular function. T1 weighted MRI revealed that the control shRNA treated tumours had numerous discrete regions with greater contrast enhancement (Fig. 5A) compared to the NG2 shRNA treated tumours (Fig. 5B). These regions corresponded to the haemorrhagic and leaky vessels observed in the histological sections. To investigate whether the alterations in vascular morphology were also accompanied by functional changes, we employed dynamic contrast enhanced MRI (DCE-MRI) and pharmacokinetic models to estimate microvascular parameters such as elimination rate of contrast agent and plasma volume fraction. Treatment with NG2 shRNAs significantly increased the transfer of contrast agent from the extracellular space to the plasma, (Kep) compared to control shRNA treated tumours (p = 0.018, t-test, Fig. 5C, D and G). Furthermore, this treatment reduced the fractional plasma volume (V_p_) in the tumour compared to control shRNAs, although this difference was not statistically significant (p = 0.17, t-test, Fig. 5E,F and H). The tracer signal intensity/time curves from the control and NG2 shRNA treated tumours (Fig. 5I) reveal four distinct phases; an initial step representing (vascular filling), slope (perfusion and permeability), maximum enhancement (leakage space) and washout. Muscle tissue was used as control in the analyses to verify that the animals received similar doses of the tracer and showed similar contrast enhancement patterns in both treatment groups (Fig. 5I). The Ktrans transfer constant, which reflects delivery of the contrast agent (by both perfusion and permeability), was higher in the NG2 shRNA treated compared the control shRNA treated tumours, although the difference was not statistically significant (data not shown).

The control shRNA treated tumours contained larger, dilated von Willebrand positive vessels compared to the NG2 shRNA treated tumours (Fig. 5J, right and left panels, respectively). Although the number of vessel elements was not significantly different, Fig. 5K, the area fraction taken up by the vessels was significantly larger in control shRNA treated tumours, (p = 0.04, t-test, Fig. 5L) indicating that they were more dilated. P13 xenografts treated with control shRNAs expressed NG2 on tumour cells but predominantly on the vessels (Fig. 5M, left panel). Treatment with NG2 shRNA reduced NG2 expression levels (Fig. 5M, right panels) although this was not statistically significant, (Fig. 5N and O). These findings indicate that targeting NG2 led to a partial normalisation of vWF positive tumour vessels, both functionally and structurally. Taken together, our findings from patient biopsy xenografts correlate with those from the cell-line based tumour xenografts, indicating that NG2 function induces rapid tumour growth, angiogenesis, increased vessel permeability and oedema, contributing to a more aggressive disease progression.

### NG2 knockdown in melanomas suppresses tumour growth and proliferation

**Figure 6:**
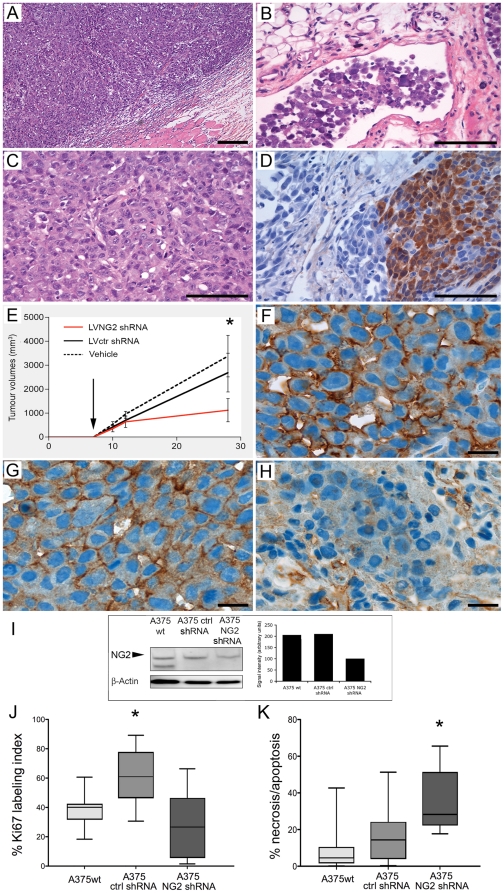
NG2 knockdown in melanomas diminishes tumour growth and proliferation. Well-demarcated, subcutaneous solid tumour (A), and lymphatic vessel infiltration (B), Solid groups of polygonal atypical tumour cells showing moderate pleomorphic nuclei with prominent eosinophilic nucleoli interspersed with connective tissue (C). Partial S-100 protein expression (D), Magnification, 200×. Growth curves of established A375 melanoma tumours treated with vehicle, control and NG2 shRNAs in *in vivo* (E, Two-Way ANOVA F_8.28_, df = 13; *p<0.0005). Arrow indicates beginning of lentiviral shRNAs or vehicle injections at day 7. Cell surface NG2 expression in parental A375 tumours (F), and in control non-functional shRNA transduced tumours (G). Reduced NG2 immunoreactivity in the NG2 shRNA transduced tumours (H), and A375 melaoma protein lysates (I). Magnification 1000×. Quantification of Ki67 positive tumour cells (J, One-way ANOVA F_9.259_; df = 3; *p = 0.0006) and cell death (necrotic/apoptotic cells by TUNEL positivity (K, One-Way ANOVA F_37.53_; df = 3; *p = 0.0001). Data represent mean ±SEM. Scale bars in A = 200 µm magnification 100×; Scale bars in B-D = 200 µm magnification 400×; Scale bars F-H = 200 µm, magnification 1000×.

Next, we validated the effect of NG2 knockdown on tumour growth in orthotopic A375 melanoma tumours with high endogenous NG2 expression levels (A375-wt). Highly demarcated solid tumours (Fig. 6A), with lymphatic vessel infiltration (Fig. 6B) were established. The tumours were composed of polygonal atypical cells showing moderate pleomorphic nuclei with prominent eosinophilic nucleoli (Fig. 6C) and S-100 expression (Fig. 6D). NG2 knockdown with NG2 shRNA significantly decreased tumour growth compared to the control shRNA treated tumours (Fig. 6E, Two-Way ANOVA F_8.28_, df = 13; p<0.0005,). At the termination of the experiment A375 melanoma tumours treated with NG2 shRNAs had a mean volume of 1120.6±486.6 mm^3^ compared to 2690±807.3 mm^3^ for those treated with control shRNA and 3375.0±866.1 mm^3^ for those injected with PBS vehicle only. Immunohistochemical analyses confirmed high NG2 expression in the untreated parental and control shRNA treated tumours (Fig. 6F and 6G, respectively). Tumour transduction with NG2 shRNAs strongly reduced NG2 protein expression (Fig. 6H and I) and decreased cell proliferation compared to the control tumours (One-way ANOVA F_9.259_; df = 3; p = 0.0006; Fig. 6J). In contrast, NG2 shRNA treatment induced greater cell death by apoptosis and necrosis that resulted in overall slower growing tumours (One-Way ANOVA F_37.53_; df = 3; p = 0.0001; Fig. 6K).

## Discussion

The prerequisite for successful translation of experimental data to clinical practice is generation and validation in relevant model systems. The present study sought to validate the role of NG2 in malignant progression and its potential as a therapeutic target using the available animal models for preclinical therapies. We showed that NG2 expression affected several aspects of tumour progression and that targeting it in GBM and melanoma xenografts suppressed angiogenesis, and reduced tumour growth. NG2 overexpression induced highly aggressive tumours characterized by increased angiogenic and moderately invasive tumour phenotypes. Indeed, larger areas with signal alterations on T2 weighted and increased contrast enhancement on T1 weighed MR images showed that the NG2 positive tumours were more rapidly growing, with disrupted blood brain barrier integrity, and increased vascular volume fraction. We have previously used two pharmacokinetic models [25], [26] in studies of NG2′s role in tumour angiogenesis[24] in order to rule out model dependency when quantifying tumour microcirculation parameters. Similar results from both methods using a high molecular weight tracer indicated that the estimated differences in transcapillary exchange between NG2-expressing compared to NG2 negative tumours were indeed due to biological differences [24]. In the present work, we used a low-molecular weight tracer that is approved for clinical use in order to increase the translational relevance. The Tofts model is the most commonly used model in both clinical and preclinical settings and is recommended for use in early-stage clinical trials [27]. However, as the Ktrans parameter is influenced by both blood flow and blood-tissue permeability, and the dominant factor is determined by the cause of the contrast agent depletion in the tissue (i.e. permeability-limited or flow-limited depletion)[26], [27].

Increased vasogenic oedema was evident in the aggressive U251-NG2 tumours that also had a more invasive phenotype compared to the U251-Wt tumours. This finding was also confirmed in the P3 and P13 GBMs, where the control shRNA treated tumours exhibited higher levels of vasogenic oedema compared to NG2 shRNA treated tumours. Oedema contributes to morbidity and mortality, and is an independent predictor of outcome for GBM patients [28]. Thus, our data and previous studies published by others collectively establish NG2 as an important factor contributing to the malignant progression.

Therapeutic strategies combining tumour and vascular targets are hypothesized to be more successful than targeting either compartment alone. As a membrane glycoprotein expressed on tumour cells and pericytes, NG2 is an attractive candidate for targeting both the tumour and angiogenic compartments within the tumour. P3 GBM xenografts expressed NG2 on both tumour cells and their angiogenic vessels and had a moderately invasive phenotype. Disruption of NG2 function in these xenografts significantly reduced tumour growth and angiogenesis by approximately 70% and 50%, respectively. However, there was no significant difference in cell proliferation and apoptosis measured at *end*-stage. These histological data and the MRI quantification showing reduced tumour volumes can be reconciled by the fact that MRI was undertaken 2–3 weeks prior to development of neurological symptoms. Indeed, three animals that did not have MRI detectable tumours were shown at post-mortem to harbour small, butterfly-like, non-angiogenic tumours. Thus, at *end*-stage tumours from both treatment groups were in an exponential growth phase characterised by rapid proliferation rates, hence the lack of statistical significance on histological analyses. Furthermore, tumour volume quantification by T1 weighted MRI alone may not always be accurate to define the tumour mass in the absence of blood brain barrier disruption and leakage of contrast agent[29]. Anti-angiogenic treatments may reduce vascular permeability to contrast agents rather exerting true anti-tumour effects[30], [31], [32]. We validated the effects on vascular normalisation in GBM xenografts derived from another patient, P13 that expressed NG2 predominantly on the highly angiogenic vessels and exhibited a less invasive phenotype. Targeting NG2 in these xenografts normalised the vasculature both structurally and functionally. The large, haemorrhagic vessels characteristic of the control shRNA treated P13 tumours became less dilated and leaky after treatment with NG2 shRNAs. The latter tumours exhibited functionally increased transcapillary re-absorption from the extracellular space to the plasma and concomitantly decreased oedema. Despite these dramatic effects on tumour angiogenesis, disruption of NG2 function by gene silencing did not result in significantly prolonged survival in neither patient derived GBM xenografts. This finding in the P3 xenografts might be explained by the presence multiple invasive tumour lesions in the brains of animals treated with NG2 shRNAs. We hypothesize that antagonisation of angiogenesis increased tumour invasiveness towards the cerebral ventricles and spread to distant sites in the brain. Previous studies demonstrated increased development of microsatellite tumours after inhibition of neo-angiogenesis by monoclonal antibodies (mAb) against vascular endothelial growth factor receptor-2 (VEGFR-2) [31], [33] [30]. The development of microsatellite tumours could be inhibited by combined treatment with mAbs against epidermal growth factor receptor [34], known to stimulate GBM migration and invasion [35]. Indeed, Bevacizumab, a humanised mAb targeting VEGF also decreases vascular permeability and increased invasion in GBM patients [36]. We showed previously that although NG2 positive tumours were highly angiogenic, they did not depend strongly on VEGF[37] but rather on diminished inhibition of angiogenesis[10]. Nevertheless, our present findings are in line with those of VEGF inhibition.

Disruption of NG2 function in the melanoma model resulted in reduced proliferation, increased apoptosis and immune infiltration by macrophages, plasma cells and histiocyte mediated granulomatous response (data not shown). Dying cells trigger inflammatory responses *in vivo*, where the ensuing hyperemia results in leakage of plasma proteins, and sustained recruitment of leukocytes and macrophages. In contrast to the melanoma tumours, NG2 shRNA treatment of both P3 and P13 GBM xenografts did not significantly reduce tumour proliferation. We postulate that this maybe due to normalisation of the angiogenic vasculature as indicated by diminished leakage of the contrast agent as detected by T1 weighted MR images. DCE-MRI with pharmacokinetic modelling revealed increased tracer efflux from the interstitium in the P13 tumours treated with NG2 shRNAs. The finding of reduced oedema, decreased fractional plasma volume fraction and area fraction of vWF positive vessels in both patient GBMs further supports this finding. As previously described, the Ktrans parameter is influenced by both tumour vascular perfusion and permeability. Vascular normalisation would increase tissue perfusion, leading to an increased Ktrans parameter as we indeed observed. Moreover, the relatively small change in the plasma volume fraction maybe due to the contribution of blood flow that masks the true plasma volume fraction in the NG2 shRNA treated tumours. Vessel normalisation also improves oxygenation. Taken together, these factors would contribute to increased tumour cell proliferation, reduced hypoxia and necrosis. Indeed, P13 tumours treated with NG2 shRNAs had significantly reduced apoptotic cells indicated by TUNEL labelling. In contrast to the GBM xenografts, NG2 shRNA treatment may have drastically reduced neovascularisation in the melanoma tumours, thereby cutting off oxygenation and nutrient supply resulting in apoptosis and arrested cell cycle progression. Although NG2 shRNAs did not prolong survival, its drastic effects on vascular normalisation might make targeting NG2 expressing cells useful as neo-adjuvant treatment in combination therapy to permit more efficient delivery of systemically administered chemotherapy drugs to the brain and to sensitise the tumours to radiotherapy (RT). Vascular normalisation would sensitise to RT by re-oxygenating the tumour. Hypoxia is known to promote radioresistance by reducing cell cycle progression, thus increased blood flow would promote tumour cell redistribution in the cell cycle and thus sensitise them to IR. Increased blood flow might also increase the bio-distribution of systemically administered chemotherapy to the tumour. Reduced oedema would also reduce tumour interstitial pressure that would prevent collapse of blood vessels and permit appropriate perfusion of therapeutic agents from the blood to the tumouŕs extracellular space. Indeed, anti-angiogenic compounds have been employed to normalize the chaotic tumour vasculature resulting in more efficient delivery of therapeutic substances to the tumour bed [32]. We showed previously that NG2 expressing tumour cells were radio- and chemo-resistant [37] [12]. Thus, targeting this cellular subpopulation might result in overall sensitisation of the residual tumour to further cytotoxic therapy.

Although gene therapy trials using lentiviral vectors at present cannot be conducted in patients due to safety and bio-distribution concerns. Delivery is a major challenge for viral gene therapy strategies to the brain. Incomplete shutdown of NG2/CSPG4 mRNA translation in the total tumour mass by the lentivirally delivered shRNA constructs, inappropriate timing of the treatment with respect to tumour growth kinetics may have contributed to the lack of survival advantage after treatment. Despite high viral multiplicity of infectious units (MOI) greater than 5×10^7^/ml, NG2 was evidently expressed on a proportion of the angiogenic vessels post shRNA transduction indicating incomplete target inhibition. Multiple injections of the shRNA constructs, even higher viral loads and reduced implanted tumour burden might enhance NG2′s inhibitory effects on tumour growth, potentiating greater survival differences. The present study utilized this method of delivery as a proof of principle approach to demonstrate that abrogation of NG2 function by gene silencing diminishes tumour growth and angiogenesis. Previous studies indicated that targeting NG2 positive tumour cells using monoclonal antibodies (mAb) conjugated to diphtheria toxin may have therapeutic potential [12], [38]. Future studies validating the combination of mAbs with adoptively transferred immune cells are currently underway to augment the anti-tumour effects of NG2 inhibition. The present study validated NG2 as an amenable therapeutic target in several relevant pre-clinical models of cancer. Since biological relevance and reproducibility are not simultaneously present in the available tumour models, we suggest that potential therapeutic targets, such as NG2, are validated in a complementary panel of pre-clinical cancer models to successfully determine their potential for clinical translation.

## Materials and Methods

### Cell culture and GBM biopsy tissue

The human glioblastoma (GBM) cell line U251N (American Type Culture Collection, Rockville, MD) was transfected with the NG2 cDNA by calcium phosphate transfection using standard protocols.The cells were propagated as multicellular spheroids in medium supplemented with geneticin to maintain transgene expression as previously described[10],[24]. U251-wt, U251-NG2 and A375 human melanoma cells (American Type Culture Collection, Rockville, MD) expressing high endogenous levels of NG2 were propagated *in vitro* as previously described [12]. The GBM biopsies were obtained from surgical resections performed at Haukeland University Hospitals. The local ethical board (REK Vest) and the Data Protection Agency in Norway approved the collection of tumour tissue. Patients gave their informed consent to specimen collection for research purposes and their samples were analyzed anonymously.

### Animals and Tumour implantation

The National Animal Research Authority in Norway approved all animal experiments. Athymic nude rats (Han: rnu/rnu Rowett) and NOD-SCID mice were used in the GBM and melanoma studies, respectively. The animals were bred in an isolation facility at 25°C (55% relative humidity) in a specific pathogen free environment and animal husbandry protocols were maintained as previously described[10]. The nude rats weighing approximately 100 g (4–6 weeks) were intracerebrally transplanted with parental U251 (U251-wt), as well as NG2 overexpressing U251-NG2 (n = 7, respectively) tumour spheroids. In addition, patient GBM biopsy spheroids (Patient 3 and 13) that had been serially *in vivo* passaged through 21 and 22 generations of nude rats (respectively) were implanted into 15 and 16 (respectively) nude rat brains as previously described [22], NOD-SCID mice (n = 35) weighing approximately 25 g (4–6 weeks) were subcutaneously injected with 5×10^5^A375 melanoma cells.

### Lentiviral vector production

Lentiviral vectors targeting NG2 were produced by subcloning the selected shRNA sequences into the pRNAt-U6.1/Lentiviral vector from GenScript (Scotch Plains, NJ). The NG2 shRNA targets the following site: 5′-GGGCUGUGUUGAAGAGUUU-3′. Target sequence of the scrambled control shRNA is 5′-GUAGAUCAAUUGGGUACACUU-3′. Lentiviral particles were produced by triple transfection of the lentiviral expression plasmids encoding shRNA, together with vsv-g encoding envelope plasmid (pMD2.G) and a gag-pol encoding packaging plasmid (psPAX.2) into HEK 293FT cells. Envelope and packaging plasmids were kindly provided by Tronolab and protocols for virus production and harvest is largely adapted from their website http://tronolab.epfl.ch. Briefly, lentiviral particles were harvested 48 h and 72 h post-transfection, sterile filtered, and concentrated for in vivo experiments by polyethylene glycol (PEG) 6000 precipitation [39]. Titration of concentrated virus stocks were performed on an Accuri C6 flow cytometer (Accuri Cytometer Inc., Ann Arbor, MI) and viral yields of ∼5×10e7 transducing Units/ml.

### Infection of primary culture monolayers by Lentiviral vectors

Monolayer cultures of primary GBM biopsy material p3 and p13 were cultivated in serum-free neurobasal medium (Invitrogen), supplemented with B-27 supplement (Invitrogen) and 20 ng/ml FGF and 20 ng/ml EGF. Viral infection was performed by centrifugation of cells in the presence of viral particles at 1200 g for 90minutes at 31°C. In vitro NG2 knockdown was validated by Western Blotting, whilst in vivo knockdown was validated by immunostaining for NG2 on tissue sections of the treated tumours. Samples of infected monolayers harvested 96 h post-infection.

### Convection enhanced delivery of shRNAs

Two-three weeks after tumour implantation, the nude rats were MRI scanned to confirm tumour engraftment. The rats were anesthetized and a craniotomy performed as previously described [22]. Viral stocks (2×10 µL) were delivered into the centre of the tumours using a glass syringe (model 701, Hamilton, Bonaduz, Switzerland) secured in a microprocessor-controlled infusion pump (UMP 2–1, World Precision Instruments, Aston, Stevenage, UK). The injection coordinates were estimated from the MRI images for each tumour. The viral shRNAs were infused by convection-enhanced delivery in the course of 25 min (200 nl/min for 10 min, followed by 400 nl/min for 10 min, and finally 800 nl/min for 5 min). 8 animals received NG2 shRNAs and 7 animals received control shRNAs against mismatched *cspg4* mRNA sequence in both P3 and P13 cohorts. After infusion, the needle was left in place for 5 min and thereafter slowly retracted and the skinfolds closed with polyamide surgical thread.

Fourteen melanoma bearing mice received intra-tumoural injections of control or NG2 shRNAs, while 7 received PBS vehicle 7 days after tumour implantation. Following surgery, the animals were monitored until recovery in an incubator at 35°C. Melanoma tumour growth was determined by measuring tumour nodules with a caliper, and tumour volumes (*V*) were calculated using the formula (*V*) = (*a*×*b*×*c*)/2, where *a, b* and *c* are the long axis, short axis and the depth, respectively, that was derived for an ellipsoid (πd^3^/6) as previously described[12].

### Magnetic resonance imaging

Animals bearing U251-NG2 (n = 3) and U251-wt (n = 4) tumours were imaged with a small animal horizontal bore 2.35T Biospec (Bruker Biospin MRI, Ettlingen, Germany) equipped with a specially designed rat head coil. T2 weighted (T2 w) spin echo (SE) images and post-contrast T1 weighted (T1 w) SE images were acquired for vasogenic oedema and solid tumour volume measurements respectively (repetition time (TR)/echo time (TE) were 500/113 ms and 524/13 ms, respectively). Both sequences were acquired using 4 NEX (number of acquisitions), matrix of 256×256 and 1 mm slice thickness. To assess microvascular status, we performed dynamic contrast enhanced MRI (DCE-MRI) using a dynamic T1 weighted SE sequence with 100 repetitions and 12 s temporal resolution in 4 axial slices (TR/TE = 91/5 ms, NEX = 2, acquisition matrix 64×64, slice thickness 3 mm). Gadolinium-based contrast agent (0.1 mmol/kg gadodiamide, GE Healthcare, Oslo, Norway) was administered in the femoral vein after baseline images were acquired, and an additional dose of 0.3 mmol/kg the contrast agent was given 4 min prior to the post-contrast T1 weighted sequence. Fifteen animals bearing P3 GBMs were scanned at 2 months, while 13 animals bearing P13 tumours were scanned two weeks after tumour implantation and 2 weeks post treatment using a 7T Pharmascan MR scanner (Bruker Biospin MRI, Ettlingen, Germany). Pre- and post-contrast T1 weighted RARE (Rapid Acquisition with Relaxation Enhancement) sequence (TR/TE = 1300/8.9 ms, NEX  = 6, acquisition matrix 256×256, slice thickness 1 mm), and a T2 weighted RARE sequence (TR/TE = 4200/36 ms, NEX = 3) were performed. DCE-MRI was performed on animals bearing human GBM from Patient 13 to assess functional changes in the microvascular status after treatment using a FLASH-sequence (Fast low angle shot) with a flip angle of 25° (TR/TE = 16.3/2.8 ms) and 550 repetitions with a temporal resolution of 1.5 s (acquisition matrix 96×96, slice thickness 1 mm). A bolus injection of gadodiamide (0.1 mmol/kg) was administered after acquisition of 30 baseline images. All sequences were obtained with identical geometric imaging parameters. The tail veins were cannulated to ensure secure injection of the contrast agent.

### MRI data analyses

Volumes of solid tumour tissue and oedema were estimated using image segmentation based on signal intensity threshold in T1 weighted post contrast and T2 weighted images, respectively, using nordicICE version 2.3.7 (Nordic Imaging Control and Evaluation, NNL, Bergen, Norway). The contrast ratio between solid tumour and healthy brain tissue in post-contrast T1 weighted images was estimated using the following equation: (SI_tumour_- SI_healthy brain_)/((SI_tumour_+ SI_healthy brain_)/2), where the SI in the healthy brain tissue was measured in the contralateral hemisphere. The DCE-MRI data were analysed by applying the pharmacokinetic model proposed by Su et al[25] and Tofts and co-workers [26]The models are based on the biological assumptions that: i) the tracer is well mixed in the plasma and ii) the blood plasma and the extracellular extravascular space (EES) are the two compartments available for the tracer. In addition, we assumed there is a bi-directional exchange between the vessels and EES. Time curves were extracted from ROIs (regions-of-interest) covering the solid tumour and discreet regions within the tumour. ROIs were delineated *a priori* based on increase in signal intensity of the tumour after contrast agent injection. When applying Su's model, the signal intensity time curves were extracted and curves from regions-of-interest (ROI) were converted to tissue concentration based on the assumption that the increase in T1 relaxation rate is proportional to the concentration of the tracer. The microvascular parameters D0 (mM, related to local blood volume), D1 (mM/min, related to blood-tissue permeability) and K_2_ (l/min, related to tissue-blood permeability) were estimated. For the Tofts model, nordicICE was used to estimate pixel-by-pixel the transfer constant, Ktrans (min^−1^, which reflects contrast delivery (by perfusion) and transport across the vascular endothelium (by permeability), with the dominant factor depending on whether the tracer depletion is flow or permeability limited), ve (fraction of extravascular extracellular space, i.e. distribution volume), vp (plasma volume fraction) and the Kep (min^−1^, which is a function of Ktrans and ve, describing the contrast agent efflux from tumour). In a previous study [24] we used both models on the same dataset in order to compare the models, where both methods gave the same results, indicating that the models were equivalent.

### MRI video

Maximum intensity projection images from T1w MR images were generated using multiplanar reconstruction and the maximum intensity projection (MIP) tool in nordicICE. Manual reorientation in multiplanar reconstructed images and MIP was recorded in real time using CamStudio™ Open Source Free Streeming Video Software.

### Histology and immunohistochemistry

Formalin fixed paraffin embedded sections from each specimen were stained with Harris Haematoxylin and Eosin (H&E, Merck, Darmstadt, Germany) according to standard procedures and examined with a Nikon Eclipse (E600) light microscope. Images were captured with a Nikon DXM1200 digital camera (Nikon Corporation, Tokyo, Japan). Indirect immunohistochemistry was performed using the EnVisionSystem, horse-radish peroxidase (HRP) and 3′3′-diaminobenzidine (DAB) (Dako, Glostrup, Denmark) method, as described previously[24]. The antibodies MIB-1 Ki67, S-100, (Dako), von Willebrand factor (Dako) and anti-NG2 were applied as described previously[24]. FFPE sections were also stained with mouse anti-rat/mouse CD8a (BD Pharmingen), mouse anti-rat/mouse CD68 (Serotec) and visualised with biotinylated horse anti-mouse secondary antibody and ABC-complex (Vectastain kit, Vector Laboratories). Double staining with CD8a (DAB) and CD68 (Liquid Permanent Red) was performed using EnVision™ G/2 Doublestain System (Dako). DAB stained tissue sections were analyzed using the image analysis system LUCIA, version 4.21 (Laboratory Imaging Ltd., Prague, The Czech Republic). The fraction of positive labelled tumour cells, defined as the Ki67 labelling index (Ki67 LI), was assessed in 5 microscopic high power fields (magnification 400×). For detection of apoptotic cells and necrosis the terminal deoxynucleotidyltransferase mediated nick end labelling (TUNEL) assay was used according to the manufacturer's instructions (Roche Applied Bioscience, Manheim Germany), with DAB as chromogen. Tunel-positive cells were assessed from the entire maximal tumour slice on low power fields in all the animals.

### Composite H&E image rendering

The rat brain sections showing maximal tumour were imaged using a Nikon TE2000 fluorescent microscope (Nikon). To cover the whole brain, 9–20 images per section were acquired using a Plan apo 2x 0.1 NA objective. Images were merged together using Adobe Photoshop CS4 extended (version 11.0.1) through the automated photomerge function.

### Statistics

The two-tailed Student's t-test was used when comparing two categories and one way- or two way-analysis of variance (ANOVA) for comparisons of more than 2 groups or variables. Mann Whitney-U test was used to analyse non-parametric data and the Kaplan Meier survival curves were analysed using the Log-rank (Mantel-Cox) test. A probability level of ≤0.05 was considered significant and all statistical analyses were performed in Graphpad Prism 5.0 software.

## Supporting Information

Figure S1 **Correlation of MRI and Histology.** Panel 1: Large, expansive H&E stained control shRNA treated tumour with pseudopalisading necrosis and shift of the midline structures. Panel 2: Small NG2 shRNA treated tumour disseminating to the contralateral hemisphere. Inserts in both panels show the area with signal change in T1 weighted images superimposed onto T2 weighted MR images.
(TIF)

Video S1 Video recorded in real time using multiplanar reconstruction and MIP from T1 weighted MRI post contrast images of control shRNA tumours demonstrating differences in tumour volumes and contrast enhancement.
(AVI)

Video S2 Video recorded in real time using multiplanar reconstruction and MIP from T1 weighted MRI post contrast images of NG2 shRNA treated tumours demonstrating differences in tumour volumes and contrast enhancement.
(AVI)
