# Supplementary figures and images for: Targeting the NG2/CSPG4 Proteoglycan Retards Tumour Growth and Angiogenesis in Preclinical Models of GBM and Melanoma
Source: PLoS One. 2011 Jul 29;6(7):e23062. doi: 10.1371/journal.pone.0023062 (PMC3146530; doi:10.1371/journal.pone.0023062)

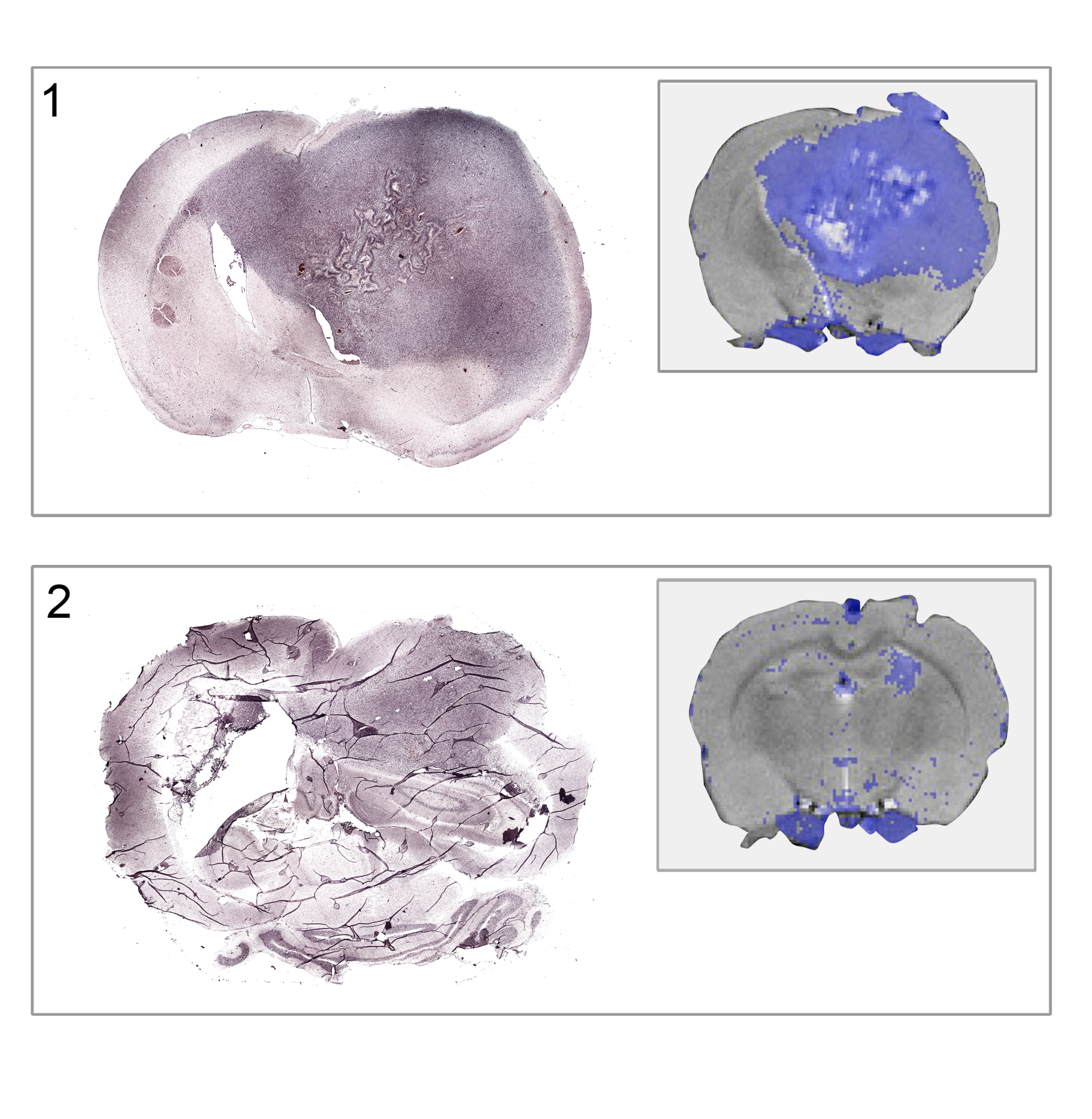

Supplement: Figure S1 — Correlation of MRI and Histology. Panel 1: Large, expansive H&E stained control shRNA treated tumour with pseudopalisading necrosis and shift of the midline structures. Panel 2: Small NG2 shRNA treated tumour disseminating to the contralateral hemisphere. Inserts in both panels show the area with signal change in T1 weighted images superimposed onto T2 weighted MR images. (TIF) [file pone.0023062.s001.tif]
